# The Self-Regulation Model of Illness: Comparison between Zika and Dengue and Its Application to Predict Mosquito Prevention Behaviours in Malaysia, a Dengue-Endemic Country

**DOI:** 10.3390/ijerph13121210

**Published:** 2016-12-06

**Authors:** Li Ping Wong, Haridah Alias, Nasrin Aghamohammadi, I-Ching Sam, Sazaly AbuBakar

**Affiliations:** 1 Department of Social and Preventive Medicine, Faculty of Medicine, University of Malaya, Kuala Lumpur 50603, Malaysia; haridahalias@gmail.com (H.A.); nasrin@ummc.edu.my (N.A.); 2 Julius Centre University of Malaya (JCUM), University of Malaya, Kuala Lumpur 50603, Malaysia; 3 Centre for Occupational & Environmental Health, Department of Social and Preventive Medicine, Faculty of Medicine, University of Malaya, Kuala Lumpur 50603, Malaysia; 4 Department of Medical Microbiology, Faculty of Medicine, University of Malaya, Kuala Lumpur 50603, Malaysia; jicsam@um.edu.my (I.-C.S.); sazaly@um.edu.my (S.A.B.); 5 Tropical Infectious Diseases Research and Educational Centre (TIDREC), University of Malaya, Kuala Lumpur 50603, Malaysia

**Keywords:** Self-Regulation Model of Illness, Zika, dengue, mosquito prevention

## Abstract

Perceptions about illnesses may influence self-care and preventive health behaviours. Illness perceptions of the Zika virus (ZIKV) infection were investigated under the framework of the Self-Regulation Model of Illness. Illness perception differences between ZIKV and dengue fever were also examined. Lastly, associations between illness perceptions of ZIKV with mosquito prevention practices were studied. Samples were drawn from landline telephone numbers using computer-assisted telephone interviewing in Malaysia. A total of 567 respondents completed the survey between February 2015 and May 2016. The median and interquartile range (IQR) for the total six dimensions of illness perceptions score was higher for dengue (23.0 (IQR 17.0–28.0)) than ZIKV (20.0 (IRQ 11.0–28.0)), *p* < 0.001. Respondents who planned to have children (OR 1.670, 95% CI 1.035–2.694 vs. no intention to have children) and had friends or acquaintances who died of dengue (OR 2.372, 95% CI 1.300–4.327 vs. no friends who died of dengue) were more likely to have a higher total score for six illness perceptions for ZIKV compared to dengue. Multivariate analysis indicated that the best predictors for mosquito control practices after the ZIKV outbreak was declared a Public Health Emergency of International Concern, in descending order, were causes, control, timeline, and consequences dimensions of illness perception. Understanding the context in which a person perceives ZIKV may contribute to developing interventions that influence prevention behaviours.

## 1. Introduction

Dengue fever is a threat to nearly half the world’s population, and to date, dengue-endemic countries are still continuously fighting the battle against *Aedes aegypti*. The new re-emergence of Zika virus (ZIKV), spread by the same mosquito vectors, has captured the world’s attention. Malaysia, a dengue-endemic country, is experiencing tremendous concern over yet another disease carried by this long existing vector. There is historical evidence of Zika in Malaysia long before the ZIKV outbreak was declared a Public Health Emergency of International Concern (PHEIC) [1]. As a matter of fact, the first isolation of ZIKV in Southeast Asia was from *A. aegypti* from Pahang, Malaysia in 1966 [2]. The existence of ZIKV was further supported by several studies that reported seropositivity of up to 30% in human samples collected in the 1950s and 1990s in East and Peninsular Malaysia [3,4,5]. Serological evidence of ZIKV in Malaysia has also been documented in monkeys and orangutans [3,6]. 

Malaysia has one of the highest numbers of dengue cases and deaths in the Southeast Asia region. In the period 2000 to 2014, the number of reported dengue cases was between 7103 and 108,698 cases per year, and the annual incidence rate ranged from 31.6 to 361.1 cases per 100,000 of the population [7]. With the historical evidence of ZIKV and the serious dengue problem in Malaysia, it is therefore clear that Malaysia is at high risk of a ZIKV outbreak. Should a ZIKV infection reach Malaysia, efforts to overcome ZIKV will be one of the nation’s greatest challenges.

Little is known about illness perception of ZIKV among the public. Since the declaration of the ZIKV as a PHEIC, Malaysian mass media has been constantly airing information from health authorities and healthcare providers on issues related to ZIKV [1], such as the facts that Zika is a milder febrile illness than dengue fever, but is more dangerous to fetuses as it causes microcephaly. Images of newborns with microcephaly are commonly aired nationwide on television. In contrast with the deadly dengue fever, there has been a flurry of media coverage on microcephaly cases. Having both diseases spread by the same vector but with different outcomes, the difference in illness perception between ZIKV and dengue is noteworthy and should be investigated. It is unclear as to what extent the surge of both local and international media has shaped the illness perception of the recently re-emerging ZIKV.

Illness perceptions are the organised cognitive representations or beliefs that patients have about their illness. Illness perception, according to the Self-Regulation Model of Illness, comprises five dimensions including (a) identity, or how much the person believes that the disease and its symptoms are threatening one’s identity; (b) timeline, or how much time the person believes that the disease will take to develop and for him/her to recover from it; (c) cause, or what the person believes may have been the reasons for the illness; (d) consequences, or how much the person realises the consequences of his/her sickness in his/her daily life, whether real or imaginary; (e) cure or control, the perception of the degree the disease can be treated or cured [8,9]. The Self-Regulation Model of Illness suggests that patients cluster their ideas about their illness by developing an understanding of the identity of the illness and symptoms, its causes, its consequences, a timeline for how long it will last, and whether it can be cured or controlled.

Cognitive and emotional representations of illness have recently attracted considerable attention, and much research has demonstrated an association between individuals’ perceptions of illness and both psychosocial and physical health outcomes. A person’s views about illness influence a number of behaviours regarding various aspects in life, including coping, adherence to treatment, and self-management behaviours [10]. The Self-Regulation Model has been examined for many illnesses and health-related behaviours including smoking behaviours regarding lung cancer [11], functioning after myocardial infarction [12], antiretroviral adherence [13], and diabetes self-management [14].

Research surrounding illness perception primarily focuses on assessing patients’ beliefs about their illness. The illness perception concept has been used to assess general public perception towards an illness and its association with important public health outcomes such as disease prevention behaviours. A recent study revealed significant associations between the illness representations of influenza A (H1N1) with H1N1 prevention behaviours [15]. In the event of the re-emergence of the ZIKV infection, conceptualising the theoretical components of the Self-Regulation Model of Illness and applying it to mosquito prevention behaviour may have a valuable role in future planning of prevention interventions. Perhaps more importantly, comparing the differences in illness perception between ZIKV infection and dengue fever may provide insights for policy makers and health authorities in revising or improving current health education and prevention messages. 

As of today, ZIKV infection from the recent outbreak has not been reported in Malaysia. Currently, little is known about the Malaysian public perception regarding the identity of illness and symptoms of the ZIKV infection, the causes and consequences of the ZIKV infection, timeline for the ZIKV infection, and whether the ZIKV infection can be cured or controlled. Evidence-based findings of the influence of the illness representation on prevention behaviour are of utmost importance. Examining these illness representation differences between ZIKV and dengue fever may provide a valuable role in planning and carrying out interventions. Guided by the Self-Regulation Model of Illness, the aim of this study is to examine (1) the differences in illness perceptions towards ZIKV infection and dengue fever; (2) the association between illness perception for ZIKV and mosquito control or prevention practices.

## 2. Materials and Methods

### 2.1. Sample

Interviews were conducted using computer-assisted telephone interviewing between February 2015 and May 2016. Sampling was drawn by random digit-dialing of landline phone numbers from all the 11 states and two federal territories in peninsular Malaysia. The selection of participants within households was accomplished by randomly requesting to speak to adults (18 years of age or older) residing in the household. Eligible participants were 18 years of age or older and had heard of the ZIKV infection. If the selected person had never heard of the ZIKV infection, the selection of respondents was accomplished by randomly requesting to speak with the next adult ≥18 years of age in the household. Only one participant per household was randomly selected to take part in the survey. Interviews were conducted between 5:30 p.m. and 10:00 p.m. on weekdays and from 12:00 p.m. to 7:00 p.m. on weekends or public holidays to avoid over-representation of unemployed participants. Unanswered calls were attempted at least two more times on separate days before being regarded as non-responses.

### 2.2. Instrument

The questionnaire comprised five sections. The first and second sections assess the participants’ socio-demographic background, their surrounding environment, and dengue experiences. The third section consisted of six questions that assessed illness perception of the ZIKV infection versus dengue fever, based on the modified concept of illness perception according to the theoretical components of the Self-Regulation Model of Illness. Instead of five dimensions, the respondents were asked to rate the perception of fear, based on the six dimensions of cognitive representations, namely, the identity of illness and symptoms, causes, consequences, timeline for how long it will last, and whether it can be cured and controlled. The additional dimension was the result of having separated the cure or control dimension, to create two separate dimensions. Participants were asked to rate their level of concern in reference to each of the dimensions in relation to ZIKV and dengue fever.

Probes providing a detailed explanation of the six dimensions of illness perceptions in relation to ZIKV infection and dengue were read out to respondents, as the following: (1) The identity component assessed the perceived fear of identity of symptoms of ZIKV infection (mild illness but leads to birth defects) compared to dengue fever (mild to severe fever but may lead to death); (2) The cause component assessed the perceived fear of a ZIKV infection caused by ZIKV, although no human cases as yet have been reported in Malaysia; in contrast, dengue is caused by the dengue virus and has long existed in Malaysia; (3) The timeline for a ZIKV infection starts with mild illness for adults and results in permanent or life-long consequences for the Zika-infected baby compared to a week of illness for dengue fever; (4) The consequences of the ZIKV infection are mild illness but infection during pregnancy, which results in birth defects. As for dengue fever, an infection may lead to a more severe illness and death in severe dengue; (5) With regards to curability, Zika infected babies are not curable in contrast to dengue fever; (6) There is little control over the ZIKV infection of a fetus, but a person with dengue fever can seek treatment to alleviate symptoms or prevent its progression to dengue hemorrhagic fever. In relation to each illness dimensions above, participants were asked on a scale of 0 (not worried at all) to 6 (worried all the time), how worried they are in relation to each of the six illness dimensions for the ZIKV and dengue fever. The response options were ‘not at all’, ‘rarely’, ‘occasionally’, ‘sometimes’, ‘frequently’, ‘usually’, and ‘every time’, scored as 0, 1, 2, 3, 4, 5, and 6, respectively, with higher scores representing a higher level of worry or concern. The score of each illness perception dimension ranged from 0 to 6. Combining all scores for the six illness perceptions gives the total six dimensions illness perception score ranging from 0 to 36, a higher total score of six dimensions illness perception representing a higher level of worry. In multivariate analysis, proportion of respondents with total score of six dimensions illness perception higher for Zika than dengue was used as a dependent variable in the multivariate logistic regression model. Individuals with a total score of illness perception higher for ZIKV than dengue was coded ‘1’. A total score of illness perception of Zika lower than or the same as dengue fever was coded ‘0’. 

The fourth section determined differences in mosquito prevention practices before and after the Zika virus infection was declared a PHEIC. Mosquito prevention practices were measured as “From the scale of 0–6, rate the general mosquito prevention practices before and after you have heard of ZIKV”. The score of mosquito control practices ranged from 0 to 6 (0 = never; 1 = rarely; 2 = occasionally; 3 = sometimes; 4 = frequently; 5 = usually; 6 = every time), with higher scores representing a higher level of mosquito prevention practices. 

The questionnaires were in three languages: Bahasa Malaysia (the national language of Malaysia), English, and Chinese. Interviews with respondents were conducted by a team of multi-ethnic enumerators; each interviewer was assigned to interview respondents of a similar ethnic group in their native language. Informed consent was obtained verbally. Respondents were assured that all responses were confidential and were reminded that completing the interview indicated voluntary participation. The study participants were not enumerated. The study was approved by the Medical Ethics Committee, University Malaya Medical Centre, Kuala Lumpur, Malaysia (MECID NO: 20162-2194).

### 2.3. Statistical Analysis

Descriptive analysis was performed to determine frequency distribution of demographic factors, perceptions, and prevention practices. The Likert scale scoring for illness perceptions and prevention practices was presented as median and interquartile range (IQR). Homogeneity of the six items of illness perceptions was assessed by calculating Cronbach’s alpha coefficient [16] and item-to-total correlations (Spearman rank correlations) between each item and the total scale. Correlations between the individual items and the total scale were calculated when the particular item was omitted from the total scale [16].

Multivariate logistic regression analyses were used to investigate factors associated with a higher total score of the six dimensions of illness perceptions of Zika infection than dengue. All significant variables (*p* < 0.05) in the univariate analysis were entered into multivariate logistic regression analysis using a simultaneous forced entry model (enter method). Odds ratios (OR), 95% confidence intervals (95% CI), and *p*-values were calculated for each independent variable. The model fit was assessed using the Hosmer-Lemeshow goodness of fit [17].

Spearman’s correlation coefficients (ρ) were used to determine the association between the score of the six dimensions of illness perceptions and the score of mosquito prevention practices after the ZIKV infection was declared a PHEIC. Multivariate linear regression was used to identify which of the six illness perceptions were the best predictor of the score of mosquito prevention practices after the ZIKV infection was declared a PHEIC. Coefficients having a *p*-value of 0.05 or less were considered statistically significant and entered into the multivariate linear regression model. 

## 3. Results

A total of 567 (70.8%) respondents completed the survey of 801 total eligible households contacted. As shown in Table 1, the majority of the respondents were female (71.1%). The ethnic majority of Malaysia, the Malays, comprised 75.1% of total respondents. The majority of respondents reported having an average monthly income of MYR2,001 to MYR4,000 (one Malaysian Ringgit was equal to USD 0.25 on date). A total of 9.3% of the study respondents had dengue fever. Approximately half of the respondents noted that dengue was a problem in their neighbourhood (51.5%), and had neighbourhood experience with dengue (49.6%).

The six items of illness perceptions of ZIKV was found to be homogenous with a Cronbach’s alpha coefficient of 0.887 and significant item-to-total correlations ranging from 0.670 to 0.938. Likewise, the six items of illness perceptions of dengue was found to be homogenous with a Cronbach’s alpha coefficient of 0.939 and significant item-to-total correlations ranging from 0.628 to 0.928. Table 2 shows a comparison of the score of the six dimensions of illness perceptions for both ZIKV infection and dengue fever. The median scores for consequences and curability dimensions were similar for both ZIKV and dengue (median 4, respectively); however, for the remaining four dimensions, the median scores were higher in dengue than ZIKV. In contrast to Zika, all of the scores for the six dimensions of illness perception for dengue were consistent at a median of 4. The consequences dimension has the highest mean score for both ZIKV and dengue.

Out of a maximum 36 score, the median (IQR) for total score of the six dimensions of illness perception of ZIKV was 20.0 (11.0–28.0), whereas for dengue it was 23.0 (17.0–28.0), *p* < 0.001. Table 1 (second column) illustrates the total score of the six dimensions of illness perception for ZIKV and dengue by demographic characteristics, dengue experiences, and the surrounding environment. Of note, the total score for the six dimensions of illness perception for ZIKV was significantly higher for females than males and for those that had friends or acquaintances who died of dengue than without. Respondents who reported that dengue was a problem in their neighbourhoods and had a neighbour with dengue fever recorded significantly higher median scores for the total six dimensions of illness perception score for ZIKV. There was a significant gradient increase in the median score for the total six dimensions of illness perception score for ZIKV virus infection along with an increase in the mosquito problem in the neighbourhood. Likewise, the median score for the total six dimensions of illness perception score significantly increases along with an increase in the frequency of fogging in the neighbourhood.

As also shown in Table 1 (column 3), of the overall respondents, a total of 22.4% (*n* = 157) had a higher total score for the six dimensions of illness perception for ZIKV than dengue, compared to 77.6% (*n* = 410) who had a total for six dimensions score of illness perception of ZIKV that was similar or lower than dengue. There were significant differences between the disparities in the total score for the six dimensions of illness perception between ZIKV and dengue, for those planning to have children among the married respondents and having friends or acquaintances who died of dengue. When these two independent variables were entered into the multivariate logistic model, the respondents who planned to have children had higher odds of having a higher total score for the six dimensions illness perception for ZIKV (OR 1.670 (95% CI 1.035–2.694)), than those who did not plan to have children. Respondents who had friends or acquaintances who died of dengue had higher odds of having a higher total score of the six dimensions of illness perception for ZIKV (OR 2.372 (95% CI 1.300–4.327)), than those without friends or acquaintances who died of dengue. The model accounted for 2.8% of the total score of six dimensions of illness perception (*R*^2^ = 0.028), and the Hosmer-Lemeshow test was not significant (χ^2^ = 181, *p* = 0.671), indicating a good model fit.

Findings of the bivariate correlation between scores of mosquito control practices after ZIKV infection was declared a PHEIC and the score of the six dimensions of illness perceptions for ZIKV are shown in Table 3. All of the scores of the six dimensions of illness perception of ZIKV were positively and significantly associated with the score of mosquito control practices after the ZIKV infection was declared a PHEIC. The highest correlation coefficients (*ρ*) occurred with the dimensions of control (*ρ* = 0.425) and causes (*ρ* = 0.394). Further analysis with a multivariate linear regression model showed that of the six illness perceptions, only causes, control, timeline, and consequences dimensions are significantly predictive of mosquito control practices after the ZIKV infection was declared a PHEIC, in order of descending beta value. The model was significant (*F*-test = 41.886, df = 6560, *p*-value < 0.001), and explained approximately 30% of the variation in the score of mosquito control practices after the ZIKV infection was declared a PHEIC. The *F*-test is highly significant, thus, this implies that there is a linear relationship between the variables in the model.

## 4. Discussion

The findings show that the score for illness perceptions for dengue were similar across all six dimensions; however, there were significant differences in scores of illness perceptions for ZIKV. Many have higher concern over the consequences and curability of Zika. This has important implications for both future education interventions as well as research. These findings suggest that in order to draw public attention or concern to the ZIKV infection, future education interventions should focus on severe consequences of ZIKV infection for unborn fetus, and that, most importantly, microcephaly has life-long consequences for which there is no treatment. Treatment merely focuses on ways to decrease the impact of the associated deformities and neurological disabilities, and affected children need lifelong care [18]. The public should also be made aware of the tremendous psychosocial impact involved in long term management of children with microcephaly. The relatively lower concerns about illness perception dimensions, namely, identification of symptoms, causes, timeline, and control warrant future research to uncover possible reasons for misconceptions that cause the relatively lower concern. Qualitative research is needed to explore the public perspectives that are necessary to have a comprehensive understanding.

In this study, a higher total score of the six dimensions of illness perception was found for ZIKV compared to dengue, which implies that the overall concern about dengue fever overrides that of the ZIKV virus. The lower level of concern for ZIKV compared to dengue is perhaps due to the fact that ZIKV is not deadly and there are no long-term effects in adults. Furthermore, the median total score of six dimensions of illness perception was only 20 out of a possible 36 indicating a moderate level of concern about ZIKV virus infection. The findings imply the need to heighten public concern over the re-emergence of ZIKV. To heighten public concern over ZIKV, it is suggested that the Malaysian public be told how easy ZIKV can spread from country to country, and the potential devastation if the ZIKV were to reach Malaysia, a tropical country, where most areas are prone to mosquito infestations. The battle against dengue is still a huge challenge in Malaysia, and should the ZIKV outbreak reach Malaysia, the country will face yet another pathogen in addition to dengue, not to mention chikungunya, which is also carried by the same *Aedes* sp.

The multivariate model exploring the predictive factors associated with a higher total score for the six dimensions of illness perception for ZIKV compared to dengue revealed that having friends or acquaintances who died of dengue was the strongest predictor, followed by planning to have children. A positive aspect of the findings was the high concern about ZIKV among those who plan to have children, and who are likely to be impacted by microcephaly. Those who have friends or acquaintances who died of dengue have seen the damage of mosquito-borne diseases and are more concerned about ZIKV. On the negative side, this implies that the people who are the least affected by ZIKV were unmoved by the outbreak. Therefore, educational approaches aimed to increase the level of concern of ZIKV across all levels of the society are warranted, especially among those who believe that they may not be impacted by microcephaly from ZIKV.

The significant correlation between the score of all six dimensions of illness perception and the score of mosquito control practices found in this study suggests that all illness representations, namely, identitication of symptoms, causes, timeline, consequences, curability and control influence a person’s mosquito control practices. Of these three illness representations, based on the linear correlation coefficient, higher level of fear over the control, causes and symptoms of ZIKV had strongest association with higher mosquito prevention practices. The outcome of multivariate linear regression suggests causes, control, timeline, and consequences dimensions of illness representation were the cognitive representations that should be emphasised in targeted interventions, in descending order of importance, to enhance mosquito prevention practices. This finding has several important implications. Firstly, a positive public response to new ZIKV outbreak was evidenced, where higher level worry of the new outbreak of ZIKV influenced higher mosquito preventions; Secondly, this study suggests that to enhance mosquito prevention practices, health messages should massively publicize the occurrence of new outbreaks of ZIKV and highlight the facts about little control over the occurrence of birth defects once a fetus has been infected. The public should also be informed of the life-long effect of ZIKV on a fetus that may require lifetime care, and the consequences of microcephaly to enhance mosquito prevention practices.

Therefore, based on the findings, messages to the public to enhance their mosquito prevention practices should firstly enhance their concerns over the causes of ZIKV. As public concern is likely derived from intensive reporting of the new outbreak, encouraging protective behaviours, it is of foremost importance that media in Malaysia continuously intensify publicity about ZIKV. Secondly, in regards to perception of control, it is important to inform the public that there is little control over the infection of ZIKV, and the effects could be irreversible for the fetus of a pregnant woman who is infected. Malaysia is a Muslim-majority country, and Islam forbids the termination of a pregnancy unless it jeopardises the mother’s life. The crude birth rate in Malaysia is very high, at 16.7 per 1000 population [19]. In 2013, there were 503,914 live births registered and 511,865 in year 2014 [19]. As for the perception of the timeline, the impact of Zika-infected babies is life-long, and care for microcephaly babies imposes financial and emotional stress. The country’s high birth rate coupled with religious viewpoints about abortion means that the public should be made aware that caring for a microcephalic child causes a tremendous burden on the health system and society. Lastly, for the perception of consequences, increasing the public perception of the serious consequences of ZIKV is vitally important. 

The findings of this study are important to enable Malaysia to better understand the public perception of an outbreak that has a high potential to occur in our country. The findings will enhance preparedness for a forthcoming ZIKV epidemic, should it reach Malaysia.

## 5. Conclusions

In the present study, illness perception of the ZIKV infection was studied under the framework of the Self-Regulation Model of Illness. A comparison between illness perception of the ZIKV infection and dengue fever was also made. This study was also the first to investigate association between illness perception of ZIKV and mosquito prevention practices. In summary, the participants exhibited a moderate level of concern for all six dimensions of illness representation for ZIKV. Overall level of concern for all the six dimensions of illness representation for dengue fever was higher than ZIKV. All six dimensions of illness representations for ZIKV were significantly correlated with mosquito prevention practices; nevertheless, the results of multivariate analysis strongly point toward the need to focus on the causes, control, timeline, and consequences dimensions, in descending order of importance, to enhance mosquito prevention practices. In conclusion, the results confirm that illness representation dimensions for ZIKV are associated with mosquito prevention behaviours.

### Limitations

This study is subjected to similar limitations faced by other telephone surveys. Firstly, being a land-line telephone survey, the study excluded households without telephones and those who use only cellular telephones. Nevertheless, the study sample closely represents the general Malaysian population distribution; Secondly, the data were self-reported and may be subject to reporting bias.

## Figures and Tables

**Table 1:** Demographic characteristics and differences between total score of six dimensions of illness perception between Zika and dengue (*n* = 567).

	Total *n* (%)	Total Score of Illness Perception *n* = 567 Range 0–36				Proportion Difference in Total Score of Illness Perception *n* (%)			Multivariate Linear Regression Model ^§^
		Zika		Dengue		Zika Higher than Dengue	Zika Is Same or Lower than Dengue		Adjusted OR (95% CI)
		Median (IQR)	*p* Value	Median (IQR)	*p* Value	(*n* = 157)	(*n* = 410)	*p* Value	
***Socio-demographics***									
Age group			0.086		0.000			0.760	
18–30 years old	150 (26.5)	19.0 (11.0–28.0)		19.0 (11.0–24.0)		44 (29.3)	106 (70.7)		
31–50 years old	197 (34.7)	19.0 (11.0–26.0)		23.0 (18.0–29.0)		51 (25.9)	146 (74.1)		
>50 years old	220 (38.8)	22.5 (15.0–29.0)		24.0 (19.0–29.0)		62 (28.2)	158 (71.8)		
Gender			0.000		0.003			0.836	
Male	164 (28.9)	18.0 (10.5–24.0)		22.5 (16.0–24.5)		44 (26.8)	120 (73.2)		
Female	403 (71.1)	22.0 (14.0–29.0)		24.0 (18.0–29.0)		113 (28.0)	290 (72.0)		
Ethnic			0.000		0.000			0.166	
Malay	426 (75.1)	22.0 (13.0–29.0)		24.0 (18.0–28.0)		128 (30.0)	298 (70.0)		
Chinese	72 (12.7)	12.0 (11.0–20.0)		16.0 (11.0–23.0)		14 (19.4)	58 (80.6)		
Indian	68 (12.0)	20.0 (16.5–24.0)		26.5 (19.5–30.0)		15 (22.1)	53 (77.9)		
Others	1 (0.2)	-		-		0 (0.0)	1 (100.0)		
Highest education attained			0.551		0.055			1.000	
Secondary and below	306 (54.0)	22.0 (14.0–29.0)		24.0 (18.0–29.0)		121 (27.8)	315 (72.2)		
Tertiary (university level)	261 (46.0)	18.5 (11.0–27.0)		23.0 (18.0–28.0)		36 (27.5)	95 (72.5)		
Occupation			0.064		0.004			0.925	
Professional & Managerial	167 (29.5)	20.0 (12.5–28.0)		23.0 (17.5–28.0)		45 (26.9)	122 (73.1)		
Manual worker	82 (14.5)	20.0 (9.0–26.0)		20.0 (12.0–28.0)		22 (26.8)	60 (73.2)		
Student	63 (11.1)	17.0 (9.5–23.0)		20.0 (12.5–25.5)		18 (28.6)	45 (71.4)		
Housewife	169 (29.8)	20.0 (11.0–29.0)		24.0 (18.0–29.0)		47 (27.8)	122 (72.2)		
Retiree	78 (13.8)	22.5 (14.0–28.0)		24.0 (17.0–30.0)		24 (30.8)	54 (69.2)		
Unemployed	8 (1.4)	24.0 (18.0–26.5)		25.0 (24.0–27.5)		1 (12.5)	7 (87.5)		
Monthly income (*n* = 536) ^‡^			0.003		0.003			0.055	
≤MYR2,000	151 (26.6)	21.0 (14.0–28.0)		24.0 (18.0–29.0)		44 (29.1)	107 (70.9)		
MYR2,001–4,000	228 (40.2)	18.0 (10.0–27.0)		23.0 (12.5–29.0)		53 (23.2)	175 (76.8)		
>MYR4,000	157 (27.7)	23.0 (15.5–29.0)		24.0 (20.0–28.0)		54 (34.4)	103 (65.6)		
Marital status			0.099		0.023			1.000	
Single	131 (23.1)	19.0 (9.5–25.5)		21.0 (13.0–28.0)		121 (27.8)	315 (72.2)		
Ever married	436 (76.9)	20.0 (12.0–28.0)		24.0 (18.0–29.0)		36 (27.5)	95 (72.5)		
Have children (*n* = 436)			0.770		0.020			0.222	
Yes	391(89.7)	21.0 (12.0–28.0)		24.0 (18.0–29.0)		105 (26.9)	286 (73.1)		
No	45 (10.3)	19.5 (14.5–28.0)		21.5 (18.0–24.0)		16 (35.6)	29 (64.4)		
Plan to have children (*n* = 436) ^†^			0.757		0.000			0.032	
Yes	102 (23.4)	20.0 (10.0–28.0)		20.0 (11.0–26.0)		37 (36.3)	65 (63.7)		1.670 (1.035–2.694) *
No	334 (76.6)	20.0 (14.0–28.0)		24.0 (19.0–29.0)		84 (25.1)	250 (74.9)		Reference
Type of house			0.002		0.000			0.928	
High rise house	87 (15.3)	12.0 (9.0–22.5)		17.5 (10.5–24.0)		26 (29.9)	61 (70.1)		
Low rise house	58 (10.2)	23.0 (13.0–29.0)		23.0 (16.0–28.0)		17 (29.3)	41 (70.7)		
Terrace/Twin	304 (53.6)	21.5 (15.0–28.0)		24.0 (19.0–29.0)		81 (26.6)	223 (72.0)		
Bungalow/Village house	118 (20.8)	23.0 (15.0–29.0)		23.0 (18.0–29.0)		33 (28.0)	85 (72.0)		
Living area			0.003		0.000			0.433	
Urban	334 (58.9)	19.0 (11.5–28.0)		23.0 (16.0–29.0)		90 (26.9)	244 (73.1)		
Suburban	157 (27.7)	20.0 (12.0–28.0)		23.0 (18.0–26.0)		49 (31.2)	108 (68.8)		
Rural	76 (13.4)	24.0 (18.0–29.0)		24.0 (20.0–33.0)		18 (23.7)	58 (76.3)		
***Dengue Experiences***									
Have had dengue			0.817		0.324			0.874	
Yes	53 (9.3)	20.0 (14.0–24.0)		24.0 (20.0–28.0)		15 (28.3)	38 (71.7)		
No	514 (90.7)	20.0 (11.0–28.0)		23.0 (17.0–29.0)		142 (27.6)	372 (72.4)		
Severe/haemorrhagic dengue			0.755		0.192			1.000	
Yes	7 (1.2 )	20.0 (13.0–23.5)		24.0 (23.0–30.0)		2 (28.6)	5 (71.4)		
No	560 (98.8)	20.0 (11.0–28.0)		23.0 (17.0–28.0)		155 (27.7)	405 (72.3)		
Household member experienced dengue/severe dengue			0.081		0.009			0.631	
Yes	107 (18.9)	22.0 (14.5–30.5)		24.0 (20.5–29.0)		32 (29.9)	75 (70.1)		
No	460 (81.1)	20.0 (12.0–28.0)		23.0 (18.0–28.5)		125 (27.2)	335 (72.8)		
Friends or acquaintances died of dengue/severe dengue			0.000		0.048			0.002	
Yes	62 (10.9)	27.0 (18.0–31.0)		24.0 (21.0–29.0)		28 (45.2)	34 (54.8)		2.372 (1.300–4.327) **
No	505 (89.1)	20.0 (11.0–27.0)		23.0 (18.0–29.0)		129 (25.5)	376 (74.5)		Reference
***Surrounding Environment***									
Dengue problem in neighbourhood			0.000		0.000			0.574	
Yes	292 (51.5)	23.0 (15.0–29.0)		24.0 (20.0–29.0)		84 (28.8)	208 (71.2)		
No/Not sure	275 (48.5)	18.0 (11.0–26.0)		21.0 (16.0–28.0)		73 (26.5)	202 (73.5)		
Anyone in neighbourhood experienced dengue/severe dengue			0.000		0.000			0.134	
Yes	281 (49.6)	23.0 (15.0–29.0)		24.0 (19.0–29.0)		86 (30.6)	195 (69.4)		
No/Not sure	286 (50.4)	18.0 (10.0–26.0)		22.0 (15.0–28.0)		71 (24.8)	215 (75.2)		
Mosquito problem in neighbourhood			0.000		0.000			0.902	
None	32 (5.6)	17.5 (9.0–29.0)		24.0 (19.0–29.0)		8 (25.0)	24 (75.0)		
Low	271 (47.8)	19.0 (10.0–27.0)		22.0 (14.5–27.0)		72 (26.6)	199 (73.4)		
Moderate	202 (35.6)	22.0 (16.0–28.0)		24.0 (19.0–28.5)		59 (29.2)	143 (70.8)		
Severe	62 (10.9)	24.5 (15.0–31.0)		28.0 (22.0–33.0)		18 (29.0)	44 (71.0)		
Mosquito fogging activities in neighbourhood			0.000		0.000			0.426	
None	83 (14.6)	11.0 (8.5–23.5)		16.0 (11.0–24.0)		66 (79.5)	17 (20.5)		
Rarely	261 (46.0)	22.5 (15.0–28.0)		24.0 (18.0–29.0)		188 (72.0)	73 (28.0)		
Occasionally	139 (24.5)	20.5 (13.0–28.0)		24.0 (19.0–28.0)		97 (69.8)	42 (30.2)		
Often	84 (14.8)	23.0 (15.0–30.0)		24.0 (20.0–33.0)		59 (70.2)	25 (29.8)		
Travel or being in dengue hotspot area			0.001		0.003			0.210	
Yes	94 (16.6)	23.0 (14.0–30.0)		24.0 (21.0–30.0)		31 (33.0)	63 (67.0)		
No/Not sure	473 (83.4)	20.0 (12.0–28.0)		23.0 (18.0–28.0)		126 (26.6)	347 (73.4)		

* *p* < 0.05, ** *p* < 0.01. ^†^ Number of participants less than total 567 participants due to no response of ‘not applicable’ response; ^‡^ USD 1 = Malaysian Ringgit (MYR) 4.2; ^§^ Multiple logistic regression analysis of total score of six illness perceptions of Zika > dengue vs. total score of six illness perceptions of Zika ≤ dengue, Hosmer and Lemeshow test; chi square = 0.181, Sig = 0.671, Cox & Snell *R* Square = 0.028, Nagelkerke *R* Square = 0.040. IQR: interquartile range; OR: odds-ratio; CI: confidence of interval; SD: Standard deviation.

**Table 2:** Comparison of score of six dimensions of illness perception between Zika and dengue, *n* = 567.

Illness Perception Dimensions	Zika Virus Infection Median (IQR) 0–6	Zika Virus Infection Mean (Mean ± SD)	Dengue Fever Median (IQR) 0–6	Dengue Fever Infection Mean (Mean ± SD)	*p* Value
Symptoms	3 (1–5)	3.11 ± 1.93	4 (3–5)	3.59 ± 1.57	<0.001
Causes	3 (1–5)	3.17 ± 1.90	4 (3–5)	3.66 ± 1.61	<0.001
Timeline	3 (1–5)	3.04 ± 1.96	4 (2–5)	3.49 ± 1.67	<0.001
Consequences	4 (2–5)	3.53 ± 1.83	4 (3–5)	3.93 ± 1.37	<0.001
Curability	4 (2–5)	3.36 ± 1.86	4 (3–5)	3.77 ± 1.50	<0.001
Control	3 (2–4)	3.11 ± 1.75	4 (3–5)	3.64 ± 1.48	<0.001

**Table 3:** Association between total score of six dimensions of illness perceptions of Zika virus infection and score of mosquito control practices after Zika virus infection was declared a Public Health Emergency of International Concern (PHEIC) (*n* = 567).

	Spearman Correlation with Score of Mosquito Control Practices		Multivariate Linear Regression ^¶^			
	*ρ*	*p* Value	b	SE (b)	*â*	*p*
Symptoms	0.385	<0.001	0.048	0.070	0.059	0.491
Causes	0.394	<0.001	0.348	0.077	0.412	0.000
Timeline	0.369	<0.001	0.166	0.064	0.204	0.009
Consequences	0.234	<0.001	−0.461	0.064	−0.530	0.000
Curability	0.310	<0.001	0.028	0.028	0.033	0.662
Control	0.425	<0.001	0.296	0.296	0.325	0.000

**^¶^** *R* value = 0.557, *R*^2^ = 0.310, adjusted *R*^2^ = 0.302, *F* value = 41.886, standard error (SE) of the estimate = 1.330, *p* < 0.001.
