# Erratum: Wong, L.P., et al. The Self-Regulation Model of Illness: Comparison between Zika and Dengue and Its Application to Predict Mosquito Prevention Behaviours in Malaysia, a Dengue-Endemic Country *Int. J. Environ. Res. Public Health* 2016, *13*, 1210

**DOI:** 10.3390/ijerph14040433

**Published:** 2017-04-19

**Authors:** Li Ping Wong, Haridah Alias, Nasrin Aghamohammadi, I-Ching Sam, Sazaly Abu Bakar

**Affiliations:** 1 Department of Social and Preventive Medicine, Faculty of Medicine, University of Malaya, Kuala Lumpur 50603, Malaysia; haridahalias@gmail.com (H.A.); nasrin@ummc.edu.my (N.A.); 2 Julius Centre University of Malaya (JCUM), University of Malaya, Kuala Lumpur 50603, Malaysia; 3 Centre for Occupational & Environmental Health, Department of Social and Preventive Medicine, Faculty of Medicine, University of Malaya, Kuala Lumpur 50603, Malaysia; 4 Department of Medical Microbiology, Faculty of Medicine, University of Malaya, Kuala Lumpur 50603, Malaysia; jicsam@um.edu.my (I.-C.S.); sazaly@ummc.edu.my (S.A.B.); 5 Tropical Infectious Diseases Research and Educational Centre (TIDREC), University of Malaya, Kuala Lumpur 50603, Malaysia

Due to an error during production, the duration of the data collection in the Abstract and Methods sections of the published paper [1] were incorrect. The correct duration of data collection occurred over a 4-month period between February and May 2016.

We apologize for any inconvenience caused to the readers by this error. The article will be updated and the original will remain on the article webpage.
